# Do You See the Writing on the Wall? What Coronary Arteries Tell About Ventricular Function∗

**DOI:** 10.1016/j.jacadv.2023.100631

**Published:** 2023-10-10

**Authors:** Spencer B. King, Matthew T. Brown

**Affiliations:** a Division of Cardiology, Emory Health System, Atlanta, Georgia, USA; b Division of Cardiology, Department of Medicine, Emory University, Atlanta, Georgia, USA

**Keywords:** coronary angiography, left ventricular ejection fraction, left ventricular function

**Figure:**
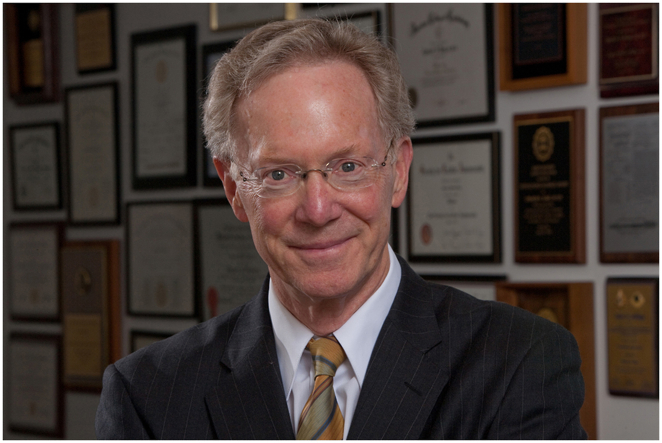

Markers written on the left ventricular wall have long given us clues to its function. Those inscriptions are the coronary arteries, which, during coronary angiography, give information about the underlying structures they serve. Experienced angiographers not only understand the distribution of the vessels and their patency or degree of obstruction but also recognize the pattern of their movement. That motion not only identifies the surface of the ventricle on which they lie but also the shortening of that surface indicating myocardial contraction. Even though these markers are primarily on the epicardial surface and in the septal structure of the heart, they can give clues to the motion below. A group of investigators at Mayo Clinic applied artificial intelligence in a deep learning (DL) approach to assess left ventricular ejection fraction (EF).

In this issue of *JACC: Advances*, Rostami et al^1^ describe a novel DL algorithm that combines a 3D-convolutional neural network (CNN) and transformer to effectively categorize EF as >40% or ≤40% based on left and right anterior oblique cine images of the left coronary artery (LCA) system during diagnostic coronary angiography. Over 18,000 coronary angiograms were used in model training (70%), validation (10%), and testing (20%) of ResNet152 3D CNN and TimeSformer Transformer video classification architectures with transthoracic echocardiography as the reference EF standard. Nearly 50% of the cases were performed for acute myocardial infarction in a predominately older male population (mean age 67 years, 35% female). In addition to the testing cohort, a randomly selected subset of 290 angiograms was also evaluated by 2 experienced interventional cardiologists for EF estimation based on coronary displacement and heart motion. The trained model analyzing both angiographic views with the combined 3D CNN and transformer achieved an area under the curve (AUC) of 0.87, sensitivity of 0.77, and specificity of 0.80, which was superior to any analysis in piecemeal. Within the dedicated cohort, comparing the DL algorithm to human observations, 2 cardiologists achieved AUC of 0.76 to 0.77, sensitivity of 0.50 to 0.44, and specificity of 0.90 to 0.93 compared to the algorithm’s respective AUC of 0.86, sensitivity of 0.75, and specificity of 0.77. With these results, Rostami et al^1^ confirmed their hypothesis that the minute displacement of epicardial coronary arteries during myocardial contraction could be processed via DL algorithms to estimate EF with as little as 2 angiographic projections of a single coronary system.

The authors propose utility of this method in the urgent cath lab situation, thereby avoiding guiding echocardiography or left ventriculography when considering the need for mechanical circulatory support. They emphasize the affordability and simplicity of integrating the DL algorithm into workflow. The group addresses several limitations including the dichotomous EF categorization, need for external validation, clinical relevance of AUC, and model transparency. Nevertheless, this early work serves as the foundation for the extraction of noncoronary data from the routine angiogram using artificial intelligence. The authors feel the study’s approach could be applied to cine images for prediction of right ventricular dysfunction, pulmonary hypertension, valvular disease, and even understudied aspects of the coronary angiogram such as vessel tortuosity and/or ectasia.

There is no doubt that there is noncoronary data to be appreciated from diagnostic angiograms. This study demonstrates the potential “fringe benefits” of incorporating artificial intelligence into a staple of cardiovascular care. As the authors described, Howard et al^2^ applied machine learning to coronary angiography in 2019 to identify catheter damping to promote patient safety and reported a sensitivity of 100% and specificity of 99%. Rostami et al^1^ have taken a different approach to incorporating artificial intelligence into invasive angiography by using it instead to gain additional diagnostic information. Estimating EF via angiography usually entails performance of left ventriculography, but this algorithm suggests interventionalists may be able to obtain more information with less contrast and radiation. However, as the authors elude, modifications of the current DL algorithm may allow for extraction of valvular information or, better yet, segmental wall motion abnormalities if left ventriculography is analyzed. Perhaps this data can be obtained by evaluating only the LCA, but just as the authors found that the full ensemble of 2 projections and 2 algorithms was better than any standalone, it makes intuitive sense that inclusion of the right coronary system and ventriculography would allow for the most complete extraction of noncoronary data by artificial intelligence.

While the test characteristics are fair in comparison to electrocardiogram-based DL models, these may be improved with analysis of additional projections, as demonstrated in the collaborative work between University of California San Fransisco and the Ottawa Heart Institute that reported an AUC of 0.91, sensitivity of 0.84, and specificity of 0.81 using all available LCA angiograms.^3^^,^^4^ This collaborative effort evaluated a significantly smaller number of angiograms—around 4,000—and categorized EF predictions not only dichotomously (>40% or ≤40%) but also as exact percentages, noting the tendency to overestimate severely reduced and underestimate hyperdynamic ventricular function. It would be interesting to know if the DL algorithm proposed by Rostami et al^1^ could better accommodate exact percentages given the larger sample size analyzed or would also fall susceptible to inaccuracies at the extremes.

Finally, as coronary computed tomography angiography becomes more widespread (and the focus of artificial intelligence itself), it begs the question of the clinical relevance of this invasive DL endeavor.^5^ While computed tomography angiography can estimate EF readily, its segmented R-R intervals for coronary assessment are no substitute for the cinematic images of angiography that capture coronary physiology throughout the full cardiac cycle. This study serves as an early proof-of-concept that there is more to be learned from angiography than the stenoses, bridging, and tortuosity that meet the naked eye. To this end, another group has recently applied artificial intelligence to invasive Doppler coronary flow reserve measurements for microvascular assessment with promising results.^6^ A potentially more impactful integration of artificial intelligence into coronary angiography would be the rapid identification of segmental wall motion abnormalities. Intraprocedural recognition of wall motion irregularities could clarify culprit vessels in cases of acute coronary syndrome when lesions are not clear or reveal viable myocardium in cases of chronic coronary disease. This group of Mayo Clinic investigators developed a DL algorithm that effectively categorizes EF based on 2 angiographic projections of the LCA during routine coronary angiography. Although we still favor left ventriculography in most cases, integration of artificial intelligence into invasive angiography emphasizes the potential physiologic and functional data that may be hiding in plain sight and can be better assessed with the aid of DL algorithms.

## Funding support and author disclosures

The authors have reported that they have no relationships relevant to the contents of this paper to disclose.
